# Pattern Switching in Soft Cellular Structures and Hydrogel-Elastomer Composite Materials under Compression

**DOI:** 10.3390/polym9060229

**Published:** 2017-06-16

**Authors:** Jianying Hu, Yu Zhou, Zishun Liu, Teng Yong Ng

**Affiliations:** 1 International Center for Applied Mechanics, State Key Laboratory for Strength and Vibration of Mechanical Structure, Shaanxi Engineering Research Center of Nondestructive Testing and Structural Integrity Evaluation, Xi’an Jiaotong University, Xi’an 710049, China; yinger.08@stu.xjtu.edu.cn (J.H.); zy2140405050@stu.xjtu.edu.cn (Y.Z.); 2 School of Mechanical and Aerospace Engineering, Nanyang Technological University, 50 Nanyang Avenue, Singapore 639798, Singapore; mtyng@ntu.edu.sg

**Keywords:** soft periodic structures, composite hydrogel–elastomer materials, pattern switching, mechanical properties

## Abstract

It is well known that elastic instabilities induce pattern transformations when a soft cellular structure is compressed beyond critical limits. The nonlinear phenomena of pattern transformations make them a prime candidate for controlling macroscopic or microscopic deformation and auxetic properties of the material. In this present work, the novel mechanical properties of soft cellular structures and related hydrogel–elastomer composites are examined through experimental investigation and numerical simulations. We provide two reliable approaches for fabricating hydrogel–elastomer composites with rationally designed properties and transformed patterns, and demonstrate that different geometries of the repeat unit voids of the periodic pattern can be used to influence the global characteristics of the soft composite material. The experimental and numerical results indicate that the transformation event is dependent on the boundary conditions and material properties of matrix material for soft cellular structures; meanwhile, the deformation-triggered pattern of matrix material affects the pattern switching and mechanical properties of the hydrogel–elastomer material, thus providing future perspectives for optimal design, or serving as a fabrication suggestion of the new hydrogel–elastomer composite material.

## 1. Introduction

Soft porous materials have been widely investigated for their remarkable mechanical properties, including isotropic foam [1,2,3] and loading-bearing cancellous bones of human shins [4]. When loaded beyond critical value, a small region or the whole structure may collapse as a result of elastic instabilities, thus leading to pattern switching and a negative Poisson’s ratio. Interest in such auxetic materials lies in the fact that a negative Poisson’s ratio may significantly improve many of the effective mechanical properties of a material (for example, the flexural rigidity and plane strain fracture toughness [5,6,7]). The research of these porous material ranges from the natural world to synthetic systems. Besides single crystals of arsenic and cadium in nature, it is found that several types of skin do show auxetic behavior, including catskin, cow teat skin, and salamander skin [8]. Shan et al. perforated a sheet with elongated cuts (arranged to form a periodic pattern with either six-fold or three-fold symmetry), to realize the 2D auxetic materials [9]. Man-made auxetic framework inspired by cubic crystals has been tailored and designed with specific Poisson’s ratios, tensile, and shear moduli [10]. Anti-tetrachiral anisotropic lattice structures with negative Poisson’s ratios have been investigated through theoretical, numerical, and experimental approaches, for their in-plane and out-of-plane elastic behaviors (which may find applications in aerospace sandwich panel cores [11]). Subsequent research models were developed for central nodules, which were connected to eight others via corner fibrils [12] or 3-, 4-, and 6-connected chiral and anti-chiral honeycombs [13], and so on.

Recently, the design of the complex microstructures in 2D lattice or 3D periodic cube structures has posed exciting challenges; increasing number of research groups are now focusing on this topic. Meanwhile, advances in fabrication technologies are enabling the realization of such special functional structures at nano- and micro-scales through the microfabrication processes, interference lithography, and 3D printing technology, where the ordered or irregular structures can easily be produced. 3D auxetic lattice via Bucklicrystal were designed and fabricated through assembling the 6-hole, 12-hole, or 24-hole units in the cubic crystal systems of the body center cubic (BCC), the solid center (SC), or the face center cubic (FCC) [14,15]. An elastic cube consisting of two sets of cylindrical voids which permeate in orthogonal directions has also been reported [16]. Exploiting the phase-transforming and switchable properties of soft materials under pressure, soft machines for actuation have been developed [17,18].

In this paper, we investigate the possibility of exploiting deformation characteristics to trigger dramatic homogeneous pattern transformations in certain classes of simple periodic elastomeric structures. Similar soft cellular structures were first studied by Bertoldi and Mullin for their mechanical properties through theory, simulations, and experiments [19,20,21,22]. There is very limited literature on the relationship between pattern transformation and material properties of the matrix material. He et al. [23] discovered that the transformation phenomenon for the shape memory polymer (SMP) can be triggered even by the stress relaxation process, through numerical simulation. In addition, Jang et al. [24] and Li et al. [25] combined pattern instability and shape-memory hysteresis of these structural materials for photonic switching. 

Apart from the research on the pattern switching of the cellular solids, another major target of this work is the hydrogel–elastomer composite material by assembling a pre-shaped cellular elastomers pattern and hydrogels together. Research on gel composites have focused on the deformation of the multilayer gel structures [26] or the fiber–reinforced hydrogels [27], toward revealing the physical mechanisms behind the designed structures. Further, surface pattern or pattern transformations can endow solid materials with some special optical, electronic, and acoustic properties due to the effects of wave interference [28,29,30]. In the present work, the hydrogel–elastomer composite material provides interesting pattern transformation and novel mechanical properties. The comparisons between the experimental and numerical results clearly show the mechanism of the pattern switch for each skeleton microstructure to be a form of local elastic instability, enabling reversible and repeatable transformation events.

## 2. Pattern Switching in Soft Cellular Solids

**Figure 1:**
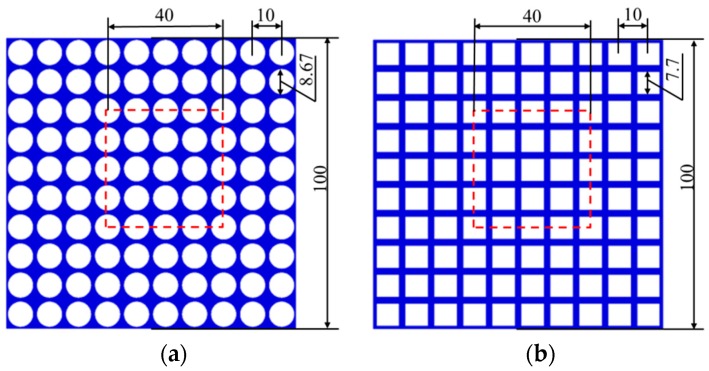
Full specimens (the whole model) and periodic representative volume elements (units in red dash line).

In order to address the issue of pattern switching in soft cellular solids, we have manufactured and performed experiments on two ordered arrays of cellular specimens which possess the geometry shown in Figure 1. Here, we explore its mechanical behavior under uniaxial compression through both experimental and numerical approaches. The size of the specimens (as shown in Figure 1a) is 100 mm × 100 mm, comprising the microstructure of a 10 × 10 mm^2^ square (arrays of circular voids of 8.67 mm diameter with 10 mm center-to-center spacing); the size of the model in Figure 1b is 100 mm × 100 mm with 10 × 10 mm^2^ square (arrays of square voids of 7.7 mm length with 10 mm center-to-center spacing). The volume fractions of voids in the two specimens are the same at 59%.

### 2.1. Materials for Soft Cellular Solids

#### 2.1.1. TangoBlackPlus^®^-3D Printing Material for the Cellular Structure

**Figure 2:**
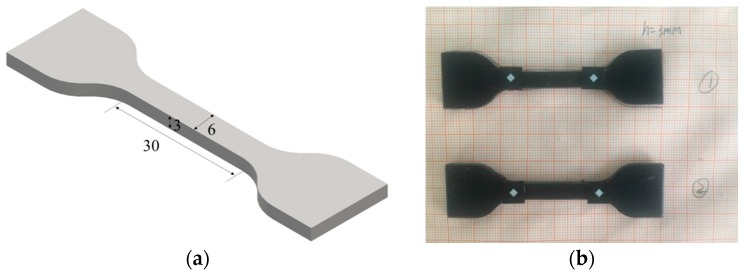
(**a**) Specimen dimensions (in mm; thickness is 3 mm) in the ASTM D638 standard, type III; (**b**) the dog-bone-shaped tensile bar for tensile testing.

**Figure 3:**
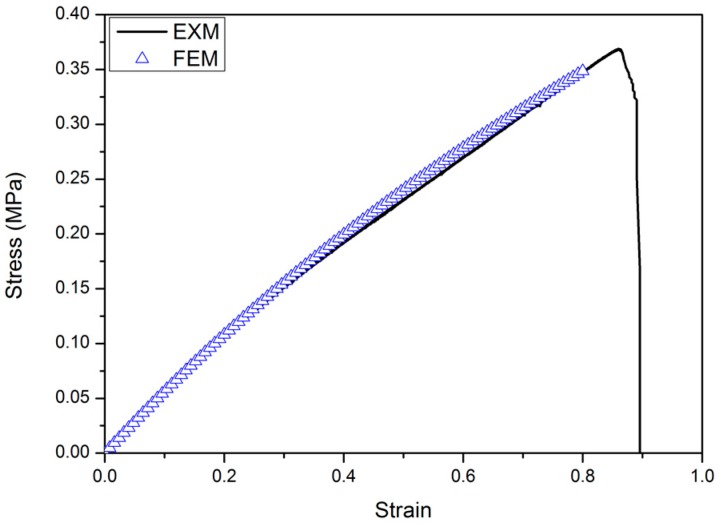
The stress-strain curve for dog-bone-shaped tensile bar—FEM (finite element method) results (open blue symbols), and experimental results (black solid line).

The first material used for the cellular structure was a 3D-printing material called TangoBlackPlus^®^ (TBP for short, Stratasys^®^ Inc., Eden Prairie, MN, USA), for which the Young’s modulus was 0.5 MPa and tensile strength was 1.2 MPa [31]. These two properties of this base material was determined by dynamic mechanical analysis (DMA). Meanwhile, the same mechanical behavior of this rubber-like material was characterized through the dog-bone-shape samples after they had been stretched in the uniaxial tension mode in a SHIMADZU (AGS-X1kN + 250 mm) testing machine (Shimadzu Corp., Kyoto, Japan). A constant strain rate of 5 mm/min was applied during loading. Dog-bone-shaped samples (refer to Figure 2)—which follow the ASTM/D638 standard (type III)—were prepared by 3D-printing technology. For simplicity, the strain and stress discussed here are the nominal strain and stress. Thus, the elastomeric stress-strain behavior can be modeled as a linear elastic model with Young’s modulus of 592.92 kPa and Poisson’s ratio of 0.44, the values of which we will use for modeling and simulation in the next Section. Figure 3 shows a comparison between the finite element method (FEM) results and the experimental output data.

#### 2.1.2. PDMS for the Cellular Structure

**Figure 4:**
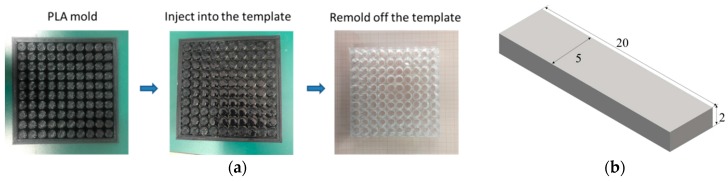
Schematic diagram of the fabrication process of the PDMS (polydimethylsiloxane) cellular structures.

We also used PDMS (polydimethylsiloxane) as the matrix material. First, we employed 3D printing technology to build a PLA (polylactic acid) mold and inject PDMS to fabricate the cellular structures (as shown in Figure 4a). The PDMS system of choice for the present process is Sylgard 184 (DOW Corning, Midland, MI, USA), which is applied in a 25.1:1 ratio (*w*/*w*) of siloxane and curing agent, and requires a thermal curing step at 70 °C for three hours. The mechanical behaviors for PDMS were characterized through the strip-shape samples (as shown in Figure 4b). Once the Young’s modulus of the PDMS is obtained through uniaxial tension testing, the elastomeric stress-strain behavior can be modeled as a compressible Neo-Hooken model with the initial Young’s modulus of 0.2 MPa and Poisson’s ratio of 0.48.

### 2.2. Experiments and Modeling for Soft Cellular Solids

#### 2.2.1. Experimental Protocol

**Figure 5:**
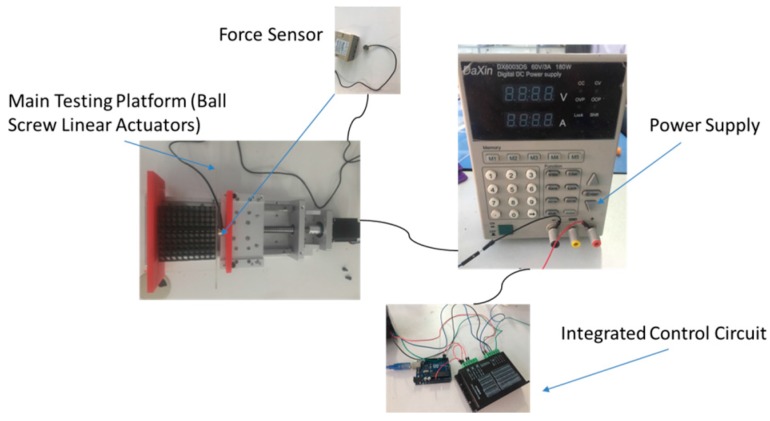
Component diagram for the handmade screw-driven testing machine.

Following the construction of the soft cellular solids (shown in Figure 1), we then used two different rubber-like materials to fabricate the continuum version of the porous periodic lattice structures. In order to achieve quantitative experimental results, we devised the test system for the soft material. The testing device can be divided into four parts: Main Testing Platform with Ball Screw Linear Actuators; Force Sensor; Integrated Control Circuit; and Power Supply (as shown in Figure 5). Each specimen was subjected to uniaxial compression in the plane of the sheet, at a constant nominal strain rate of 1 mm/min using a handmade screw-driven testing machine. The nominal stress vs. nominal strain behavior was recorded, based on computed data from the measured force and displacement.

#### 2.2.2. Modeling

Using the commercial software ABAQUS (Dassault Systèmes Simulia Corp., Providence, RI, USA), the cellular structures were simulated through two approaches: first, by considering the full specimen of dimension 100 mm × 100 mm thus capturing the geometry differences at the boundaries as well as the displacement applied on top surfaces as force, while the displacement on the bottom surface was constrained in the same direction; and secondly, by considering a representative volume element (RVE) of the domain with appropriate periodic boundary conditions and containing small internal defects in the mesh to aid the initiation of instabilities.

Numerical simulations of the deformation of the two periodic structures were conducted utilizing nonlinear finite element analysis (we represented the solids with C3D8 elements). For TBP material, the elastomeric stress-strain behavior was modeled as a linear elastic one with Young’s modulus of 592.92 kPa and Poisson’s ratio of 0.44 (the elastomeric stress-strain behavior of the PDMS material can be modeled as a compressible Neo-Hooken model, with the initial Young’s modulus of 0.2 MPa and Poisson’s ratio of 0.48). To obtain nominal stress vs. nominal strain behavior, the total force on load edge (top surface) was monitored as a function of the applied displacement.

### 2.3. Results and Discussion for Soft Cellular Solids

**Figure 6:**
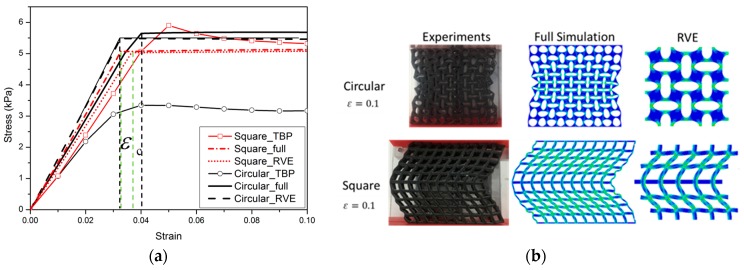
The mechanical behavior for TangoBlackPlus^®^ cellular structures is as follows: (**a**) Nominal stress vs. nominal strain curves (red solid line with open symbols and black solid line with open symbols, respectively, represent TBP with square voids and circular voids. Red dot dash dot line and red dot line depict the full model and RVE simulations for the TBP model with square voids; black solid line and black dash line show the full model and RVE simulations for the TBP model with circular voids); (**b**) Experimental images of structures at a macroscopic strain of 0.1.

**Figure 7:**
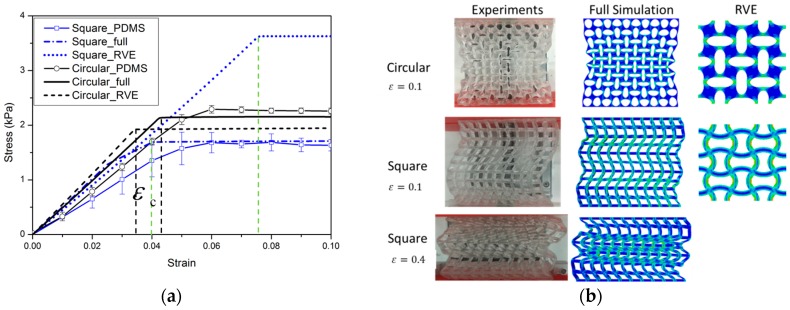
The mechanical behavior for PDMS cellular structures: (**a**) Nominal stress vs. nominal strain curves (blue solid line with open symbols and black solid line with open symbols represent PDMS cellular structures with square voids and circular voids. Blue dot dash dot line and blue dot line depict the full model and RVE (representative volume element) simulations for the PDMS model with square holes; black solid line and black dash line still show the full model and RVE simulations for the PDMS model with circular voids); (**b**) Experimental images of structures at a macroscopic strain of 0.1 and 0.4.

Experimental and numerical results for the nominal stress vs. nominal strain behavior of specimens of two types of materials (up to a compressive strain of 0.1) are shown in Figure 6 and Figure 7. The results shown provide a typical characteristic of a stress-strain response and observed pattern transformation for TBP and PDMS, respectively.

It can be seen in Figure 6a and Figure 7a that a plateau occurs in the stress-strain curve when the strains reach above ε_c_, i.e., the total stress will become independent of strain after the critical strain ε_c_ From the stress vs. strain curves, it can be observed that regardless of the kind of material property, the stress of the cellular material is approximately proportional to the strain for small displacements, and compression within the material manifests as foreshortening of interstitial vertical ligaments in the structure. The departures from linearity are the result of elastic instabilities in the microstructures that trigger the pattern transformations.

Interestingly, after the transformation to the new configuration, various patterns show different mechanical properties in their cellular structures. In the structure with ordered circular voids the mode of deformation is characterized by a critical eigenmode consisting of mutually orthogonal ellipses. The simulations for RVE and full specimen are both consistent with the experiments. As shown in Figure 6a and Figure 7a, if switching the pattern of the RVE model is the same as the full model calculation, it is clearly observed that the RVE results exhibit an earlier departure from linearity than the full model calculation, since the former does not capture the influence of the boundary conditions on the initiation of the instability. In the structure with ordered square voids, the phenomenon is totally different in that much of the macroscopic deformation is observed to be accommodated by the critical buckling of the side struts. For the elastic range in Figure 6, the images for RVE and the full specimen simulations match well with the experimental results. Only the experimental result of the TBP specimen with circular voids does not match well with the full model and RVE simulation results; the reason for this discrepancy is that compression testing leads to the fracture of the inter-hole ligaments in the matrix material. However, for the hyperelastic range in Figure 7, the RVE results capture a transformation which is a totally new and different pattern (triggered by the rotation of the matrix domains diagonally bridging neighboring hole), which thus results in the maximum critical force to pattern switching. Hence, the experimental and numerical results indicate that the transformation event is highly dependent on the boundary edge conditions and material properties of the matrix material.

## 3. Novel Mechanical Behavior of the Composite Gel Material under Compression

**Figure 8:**
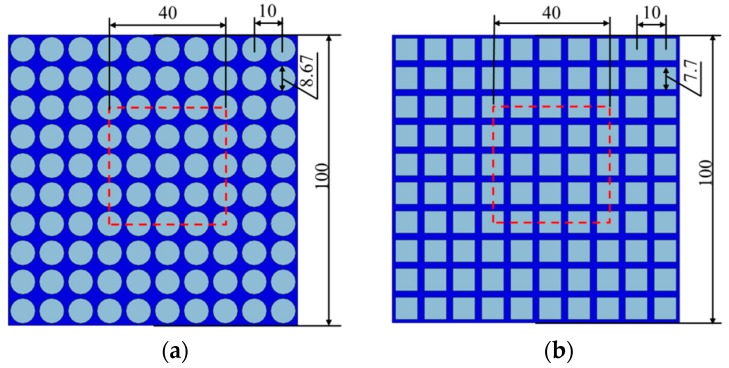
Schematic illustration for full specimens (the whole model) and periodic representative volume elements (units in red dash line) for hydrogel–elastomer composites.

The composite material that is composed of two components (hydrogel and elastomer) has been proposed in our previous work [32]. In the earlier study, we can easily understand that if the holes are filled with a more rigid material than that of the matrix, the cellular structure will not lead to any pattern switch [33]. Therefore, a much softer material needs to be placed in the holes to achieve the pattern switch for a composite material. Mullin et al. [34] discovered that the inclusion which has a Young’s modulus of greater than 1% of the bulk would suppress the pattern switch. Based on present assumptions and finite element analysis (FEA), we propose here to use two kinds of hydrogel-elastomer composite material which experimentally exhibit the pattern switch in composite gel material. In the modeling and the experiment, the specific combinations of the two materials are illustrated in Figure 8. The specimen shown in Figure 8a is a 100 mm × 100 mm square model of circular gel inclusions of 8.67 mm diameter with 10 mm center-to-center spacing in both vertical and horizontal distributions; the specimen shown in Figure 8b is 100 mm × 100 mm model with 10 × 10 mm^2^ square arrays of gel inclusions of 7.7 mm length. The volume fractions of gel in the two types of composite materials have the same percentage of 59%.

### 3.1. Material Characterization of Polyacrylamide Hydrogel

In the composite material, two different materials are used for the matrix, i.e., the elastomer for the matrix and the hydrogel for inclusions. We have already discussed the material properties of matrix material in Section 2.1. The inclusion that adheres with the matrix material is polyacrylamide hydrogel. For an ideal elastomeric gel, the effect of mixing the polymer and the solvent is represented by the osmotic pressure as a function of the swelling ratio J. We prepared the polyacrylamide hydrogels using two different procedures. For the first one (in order to obtain robust hydrogel–PDMS interfaces), acrylamide was dissolved in distilled water to 1.9 M concentration, along with *N*,*N*-methylenebisacrylamide (MBAA, 0.001× weight of acrylamide) as a crosslinker (we used α-Ketoglutaric acid (0.041× weight of acrylamide) as light-induced polymerization initiator). The gels were cured for 2 h under UV irradiation. Using the other approach (to obtain weak hydrogel–TBP interfaces), the same concentration of acrylamide and MBAA-to-acrylamide ratio was used to form gels with ammonium persulfate (0.0079× weight of acrylamide) as polymerization initiators, and *N*,*N*,*N*′,*N*′-tetramethylethylenediamine (0.0025× weight of acrylamide) as a crosslinker. The gels were cured for 2 h at a temperature of 60 °C.

It is known that the experimentally determined stress-stretch curves of polyacrylamide gels fit the Gaussian-chain model well. The free energy function for the hydrogel takes the form of the Flory–Huggins model by Hong et al. [35],

(1) $$W=\frac{1}{2}NkT(I-3-2\log J)-\frac{kT}{\nu }\left[\left(J-1\right)\log\frac{J}{J-1}+\frac{\chi }{J}\right]$$

where *N* is the number of polymeric chains per reference volume, χ the dimensionless parameter of the enthalpy of mixing, and *kT* the temperature in the unit of energy [36]. A representative value of the volume per molecule is ν = 10^−28^ m^3^, so *kT*/ν = 4 × 10^7^ Pa. Thus, a gel is fully characterized by a scalar *NkT*, and the isotropic stretch λ_0_ = *J*_0_^1/3^ for the initial free-swelling state. According to Li’s experimental results [37], the two dimensionless material properties of freely swollen gels (*N*ν and χ) can be appropriately chosen for the numerical simulation. Here we adopt the values *N*ν = 0.001 and χ = 0.5.

### 3.2. Weak or Robust Interface of the Hydrogel–Elastomer Composite Material

**Figure 9:**
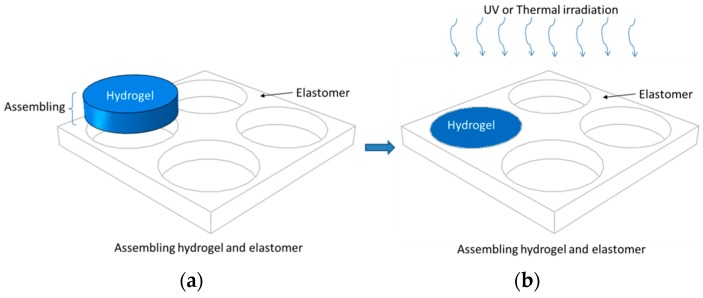
A schematic illustration of the fabrication of robust micro-structured hydrogel–elastomer composite material: (**a**) The pre-shaped hydrogel and elastomer are assembled together; (**b**) After ultraviolet or thermal irradiation, the resultant hydrogel composite material forms.

In Section 3.1, we have mentioned two approaches to assemble pre-shaped patterned cellular elastomers and hydrogels together for the fabrication of hydrogel–elastomer composite material, as shown in Figure 9.

**Figure 10:**
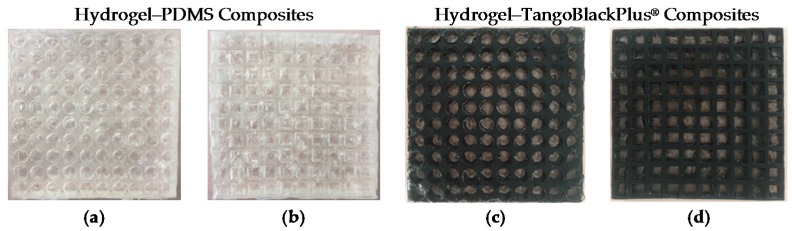
Specimens of hydrogel–elastomer composite material fabricated through ultraviolet (**a**,**b**) or thermal (**c**,**d**) irradiation.

To obtain robust interfaces for composite hydrogel–PDMS solids, we adopt the simple yet versatile method proposed by the Zhao group [38]. The key point is to address PDMS’ oxygen inhibition. We spray the acetone solution of diphenyl ketone on the elastomer surface and use an ethanol solution to wash it several times for surface absorption of the benzophenone solution. The benzophenone acts as an ultraviolet-assisted grafting agent for covalent crosslinking hydrogel polymers on elastomer surfaces. The assembled composite material is exposed under ultraviolet irradiation together with the synthesis of polyacrylamide hydrogel. The hydrogel–TangoBlackPlus^®^ composite material can be simplified by filling the pre-shaped hydrogel into the porous material, which means that there is no bonding between the matrix material and inclusions. This is mainly due to the elastomer surfaces’ inhibition of polymer crosslinking and grafting, as well as fluidic characters of pre-gel solutions (which will block the interfacial connection if no special chemical treatment is carried out). The hydrogel–elastic composite materials fabricated are illustrated in Figure 10.

### 3.3. Results and Discussion of Composite Material

**Figure 11:**
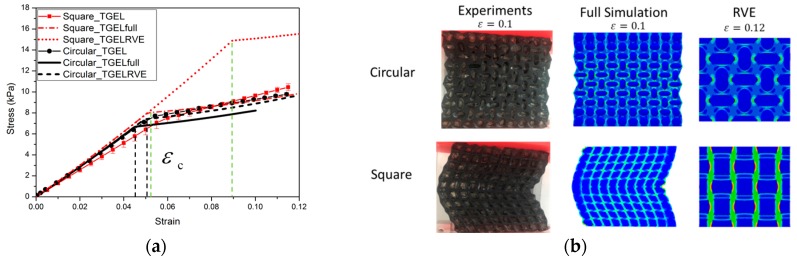
The mechanical behavior for hydrogel–TangoBlackPlus^®^ composites: (**a**) Nominal stress vs. nominal strain curves (red solid line with full symbols and black solid line with full symbols, respectively, represent hydrogel–TBP composites with square inclusions and circular inclusions. Red dot dash dot line and red dot line depict the full model and RVE simulations for hydrogel-TBP model with square inclusions; black solid line and black dash line show the full model and RVE simulations for hydrogel-TBP model with circular inclusions); (**b**) Experimental images of specimens from experiments and full model simulation result at macroscopic strain of 0.1, pattern transformation of RVE model at macroscopic strain of 0.12.

**Figure 12:**
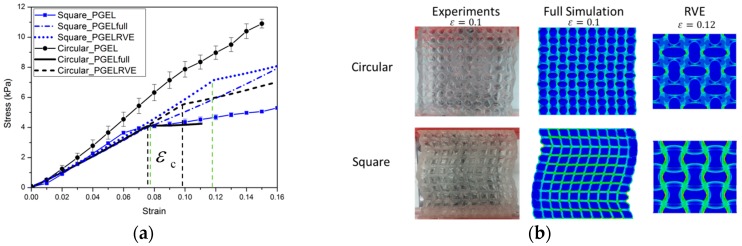
The mechanical behavior for hydrogel–PDMS composites: (**a**) Nominal stress vs. nominal strain curves for hydrogel–PDMS composites specimens (blue solid line with full symbols and black solid line with full symbols respectively represent hydrogel–PDMS composites with square inclusions and circular inclusions. Blue dot dash dot line and blue dot line depict the full model and RVE simulations for hydrogel–PDMS model with square inclusions; black solid line and black dash line show the full model and RVE simulations for hydrogel–PDMS model with circular inclusions); (**b**) Experimental images of specimens from experiments and full model simulation result at macroscopic strain of 0.1, pattern transformation of RVE model at macroscopic strain of 0.12.

When a periodic elastomeric cellular solid is compressed, the array of pores undergoes an unstable transformation at a critical point [39,40,41,42]. Similar instability processes also trigger the change to new configurations in the novel composite gel materials [32,34]. The experimental and numerical results capture the mechanical behavior of hydrogel–elastomer composite material, as shown in Figure 11 and Figure 12.

As expected, the mechanical behavior of the hydrogel–elastomer composite material displays almost linear elastic behavior at the initial stage with a change to a different elastic relationship at the critical strain of ε_c_. The patterns for the composite materials at the nominal strain of 0.1 are shown in Figure 11b and Figure 12b. From Figure 11a, the mechanical properties of the hydrogel–TBP composite material in the experiments match well with the numerical analysis. However, the experimental results for hydrogel–PDMS composite material are not consistent with numerical analysis. The reason is that material property of PDMS has changed when it was exposed to UV irradiation [43,44]. It should be noted that this often occurs in experiments and should be taken into consideration in future research simulation work.

**Figure 13:**
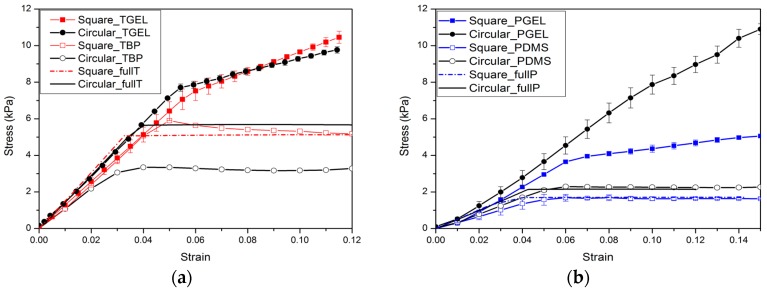
(**a**) Comparison between the TBP (TangoBlackPlus^®^) cellular structure and hydrogel-TangoBlackPlus^®^ composites (red solid line with full symbols and black solid line with full symbols respectively represent hydrogel–TBP composites with square inclusions and circular inclusions; the red solid line with open symbols and the black solid line with open symbols respectively represent the TBP cellular structure with square voids and circular voids; the red dot dash dot line and the black solid line depict the full model simulation for the TBP model with square voids and circular voids); (**b**) Comparison between the PDMS cellular structure and the hydrogel–PDMS composites (blue solid line with full symbols and black solid line with full symbols, respectively, represent hydrogel–PDMS composites with square inclusions and circular inclusions; the blue solid line with open symbols and the black solid line with open symbols, respectively, represent the PDMS cellular structure with square voids and circular voids; the blue dot dash dot line and the black solid line depict the full model simulation for the PDMS model with square voids and circular voids).

Figure 13 also provides a comparison between the matrix materials and hydrogel–elastomer composite material. In stress vs. strain curves, soft cellular material and hydrogel–elastomer composites both exhibit elastic properties before the pattern switching. Following pattern transformation, the stress plateaus emerge in soft cellular material; while the stress is dependent on strain in hydrogel–elastomer composites with the inclusions plugging. It is noted that the deformation-triggered patterns of the composite materials depend on the deformed pattern of the matrix material; while the gel inclusion can temporarily repair fractured ligaments and cracks on the matrix material. 

**Figure 14:**
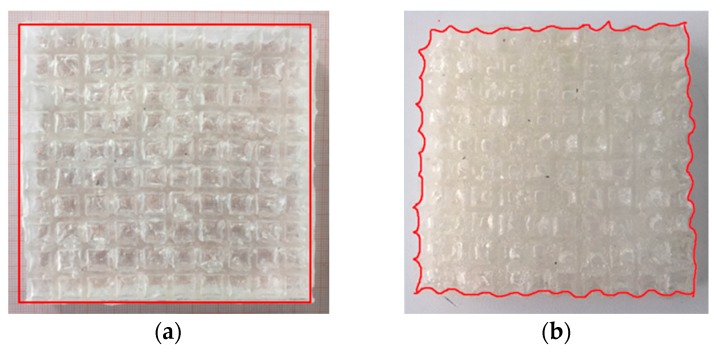
Hydrogel–PDMS composite material buckles with the deswelling of the gel inclusions at room temperature.

In the case of hydrogel–PDMS composite material (shown in Figure 14a), the material is again deformed because there is covalent crosslinking between the PDMS and hydrogels in the robust interface. As the gel inclusions deswell at room temperature, the side of the specimen buckles and the buckling mode is evident in Figure 14b. As there is no covalent attachment between TBP and the hydrogel, the phenomena thus did not exist in hydrogel–TBP composites.

## 4. Conclusions

The aim of this paper was to progress the investigations into the mechanical properties of soft cellular material and the associated designed composite material through experimental and numerical analyses. We first investigated the deformation-triggered patterns on soft cellular materials with different material properties. The experimental and numerical results indicate that the transformation event is highly dependent on the boundary edge conditions and material properties of the matrix material.

Next, we fabricated novel hydrogel–elastomer composite materials using two approaches by assembling the gel inclusions into the periodic elastomeric cellular structures under ultraviolet or thermal irradiation. From our experiments, we fortuitously found that gel inclusion can temporarily repair fractured ligaments and cracks on the matrix material, such that the initial rupture of the matrix material does not affect the mechanical properties of the hydrogel–elastomer material. This finding may provide future perspectives for optimal design or serve as a fabrication suggestion for new gel composite materials.
